# Simple Clinical and Laboratory Predictors of Chikungunya versus Dengue Infections in Adults

**DOI:** 10.1371/journal.pntd.0001786

**Published:** 2012-09-27

**Authors:** Vernon J. Lee, Angela Chow, Xiaohui Zheng, Luis R. Carrasco, Alex R. Cook, David C. Lye, Lee-Ching Ng, Yee-Sin Leo

**Affiliations:** 1 Department of Clinical Epidemiology, Tan Tock Seng Hospital, Singapore, Singapore; 2 Saw Swee Hock School of Public Health, National University of Singapore, Singapore, Singapore; 3 Department of Statistics and Applied Probability, National University of Singapore, Singapore, Singapore; 4 Department of Biological Sciences, National University of Singapore, Singapore, Singapore; 5 Program in Health Services and Systems Research, Duke-NUS Graduate Medical School, Singapore, Singapore; 6 Department of Infectious Diseases, Tan Tock Seng Hospital, Singapore, Singapore; 7 Environmental Health Institute, National Environment Agency, Singapore, Singapore; Centers for Disease Control and Prevention, United States of America

## Abstract

**Background:**

Dengue and chikungunya are co-circulating vector-borne diseases with substantial overlap in clinical presentations. It is important to differentiate between them during first presentation as their management, especially for dengue hemorrhagic fever (DHF), is different. This study compares their clinical presentation in Singapore adults to derive predictors to assist doctors in diagnostic decision-making.

**Methods:**

We compared 117 patients with chikungunya infection diagnosed with reverse transcription-polymerase chain reaction (RT-PCR) with 917 dengue RT-PCR-positive adult patients (including 55 with DHF). We compared dengue fever (DF), DHF, and chikungunya infections by evaluating clinical characteristics of dengue and chikungunya; developing classification tools via multivariate logistic regression models and classification trees of disease etiology using clinical and laboratory factors; and assessing the time course of several clinical variables.

**Findings:**

At first presentation to hospital, significantly more chikungunya patients had myalgia or arthralgia, and fewer had a sore throat, cough (for DF), nausea, vomiting, diarrhea, abdominal pain, anorexia or tachycardia than DF or DHF patients. From the decision trees, platelets <118×10^9^/L was the only distinguishing feature for DF versus chikungunya with an overall correct classification of 89%. For DHF versus chikungunya using platelets <100×10^9^/L and the presence of bleeding, the overall correct classification was 98%. The time course analysis supported platelet count as the key distinguishing variable.

**Interpretation:**

There is substantial overlap in clinical presentation between dengue and chikungunya infections, but simple clinical and laboratory variables can predict these infections at presentation for appropriate management.

## Author Summary

Dengue and chikungunya are mosquito-borne diseases that are found in similar geographical areas and present with similar symptoms. As their treatment is different, especially for dengue haemorraghic fever (DHF) which is a more severe form of dengue, it is important for healthcare workers to differentiate between them. We studied 117 chikungunya and 917 dengue adult patients (including 55 with DHF) by comparing their clinical presentation and developed decision trees to classify them using simple symptoms and laboratory tests. From the study, we found that at their first appearance in hospital, more chikungunya patients had muscle or joint pains, and fewer had a sore throat, cough, nausea, vomiting, diarrhea, stomach pain, loss of appetite or fast heart beat than DF or DHF patients. From the decision trees, of DF versus chikungunya using only platelet level cut-offs, we could correctly classify 89% of the cases. For DHF versus chikungunya using platelet level cut-offs and the presence of bleeding, the correct classification was 98%. The use of these simple decision trees can therefore predict the subsequent development of these infections for appropriate treatment.

## Introduction

Dengue and chikungunya are vector-borne diseases that have been circulating in the tropical regions of Africa and Asia for decades [1], [2]. Many factors influence the geographical spread of both viruses, including vector distribution (both are spread by *Aedes* mosquitoes), human travel, urbanization, and climatic changes [1], [2]. These two diseases now co-circulate in many countries [3], [4] and pose a challenge to clinicians because they may require different clinical management even though their manifestations can be similar. Dengue fever (DF) cases can develop into severe dengue [5], dengue hemorrhagic fever (DHF) or dengue shock syndrome (DSS) [6], which may lead to adverse outcomes including death, especially in children. A previous Singapore study found that of the DF cases presenting to hospital, 6% subsequently developed DHF and 0.5% DSS [7]. The possibility of these complications necessitates early identification, close monitoring for plasma leakage and possible institution of fluid therapy for dengue cases [5], [6], [8]. At the same time, a substantial proportion of dengue cases are mild and do not require hospitalization – only regular outpatient monitoring and symptomatic treatment [7], [9]. Most chikungunya cases do not result in severe complications and treatment is symptomatic unlike DHF or DSS cases, although atypical presentations sometimes occur including organ failures which can be fatal especially in elderly with co-morbidities [10].

The classical manifestations of these two diseases have substantial overlap with a substantial proportion of both diseases having fever, headache, myalgia, and rash [3], [4], [6]. Of some of the studies comparing the presentations of dengue and chikungunya – shorter duration of fever, connjunctivitis, acute arthritis, myalgia/arthralgia and rash were more prominent in chikungunya [11]–[17]; while leukopenia, neutropenia, thrombocytopenia and abdominal pain [13]–[17] were more prominent in dengue cases.

Although knowing the diagnosis of dengue and chikungunya cases early is important, in resource-limited settings, sophisticated laboratory tests to distinguish these infections may be unavailable or costly, necessitating epidemiological and symptom-based approaches to diagnosis.

In Singapore, all four dengue serotypes co-circulate and the number of cases and occurrence of major outbreaks have increased since the 1990s, despite an effective vector control program since the 1970s [18], [19]. Unlike many other Southeast Asian countries where dengue is primarily a pediatric disease, most notified infections are in adults in Singapore [18]. Although outbreaks of chikungunya were recorded in South and Southeast Asian countries since the 1960s, indigenous transmission of chikungunya in Singapore was only reported in 2008 after the wave of Indian Ocean outbreaks starting in 2005 [20], [21].

This study compares the clinical manifestations of dengue and chikungunya in adults in Singapore, and aims to derive predictors of chikungunya versus dengue in the presence and absence of laboratory tests, to assist doctors in both well-resourced and resource-limited settings in diagnostic decision-making. It builds on our previous studies performed in Singapore on a 2004 dengue cohort to derive predictive algorithms for DHF for doctors to determine need for hospitalization among patients presenting with dengue [7], [22].

## Methods

We conducted a retrospective case-control study on 117 patients confirmed with chikungunya infection on reverse transcription-polymerase chain reaction (RT-PCR) during the August 2008 outbreak, and hospitalized at Tan Tock Seng Hospital, Singapore, the national outbreak response center. These were compared with 917 dengue-PCR positive adult patients (including 55 with DHF) hospitalized at the same center during the large 2004 dengue outbreak in Singapore. The methods and performance of the dengue [23] and chikungunya [24] PCR tests we used have been previously documented and used for routine diagnosis. Specific information on possible co-infections of chikungunya cases in this cohort was not available. However, the risk of co-infection was low, demonstrated by tests of 900 chikungunya-positive cases from January to August 2008 for dengue at the Environmental Health Institute, Singapore, which yielded no evidence of co-infection. This is likely due to the fact that the chikungunya outbreak in August 2008 was driven by *Aedes albopictus* in less-urbanized parts of Singapore, [24], in contrast to dengue which is usually transmitted by *Aedes aegypti* in highly urbanised areas [18].

For the two cohorts, medical records were reviewed for all hospitalized patients during the study periods (31 July to 11 November 2008 for chikungunya, and 1 January to 31 December 2004 for dengue) with positive chikungunya and dengue PCR results respectively. Demographic, epidemiological, serial clinical and laboratory, radiological, treatment and outcome data were extracted and anonymized. In Tan Tock Seng Hospital, dengue patients are managed using a standardized care path which ensures that clinical and laboratory data are uniformly observed. A similar care path was developed for chikungunya, ensuring that similar clinical data were collected. The criteria for DF, DHF and DSS are in accordance with the World Health Organization 1997 dengue guideline [6].

### Statistical analyses

We performed three analyses comparing dengue and chikungunya infections. In all three, DF and DHF (including DSS) were treated separately, i.e. DF and chikungunya were compared to provide clinicians with an understanding of key differences between the two diseases with similar expressions, while DHF and chikungunya were contrasted because DHF requires additional clinical management. In the first of the three analyses, we compared clinical characteristics of dengue and chikungunya using univariate chi-squared and Wilcoxon tests to quantify differences between diseases for dichotomous and continuous variables, respectively, using data from the entire hospitalization. In the second analysis, we developed classification tools via multivariate logistic regression models and classification trees of disease etiology using clinical and laboratory factors that might guide clinical decisions before laboratory confirmation was available: we therefore only used data available at the time of hospital presentation, considering clinical data only for resource-limited settings, and clinical and laboratory data for well-resourced settings. In the third analysis, we assessed the time course of several clinical variables using Bayesian hierarchical modelling.

### Multivariate logistic regression

The data exhibited separation [25], [26] which prevented finite estimates of (adjusted) odds ratios and consequently had deleterious effects on Wald-derived confidence intervals [25]. To address this, we used Firth's modified score procedure to estimate odds ratios and derived confidence intervals using the profile-penalized likelihood function [26]. We fit a multivariate model, i.e. accounting for confounding, with the following variables: age, gender, hypertension, time since onset (in days), duration of fever (in days), presence of fever, headache, myalgia/athralgia, rash, any bleeding, sore throat, cough, nausea, vomiting, diarrhea, abdominal pain, anorexia, maximum temperature (°C), tachycardia (pulse >100/minute), leukocyte count, hemoglobin, serum hematocrit, platelet count, lymphocyte/neutrophil/monocyte/atypical lymphocyte proportion, serum sodium, potassium, urea, creatinine, bilirubin, alanine (ALT) and aspartate aminotransferase (AST), alkaline phosphatase (ALP), protein and albumin, as measured on the day of hospital presentation. Missing values for continuous variables were imputed using the mean over all non-missing records. Terms not statistically significantly different from 0 were removed from the multivariate model sequentially, starting with the one with the highest *p*-value. In secondary analysis, we extended the time horizon to the entire duration of hospitalization, replacing laboratory variables with either maximum or minimum recorded value depending on clinical significance.

### Classification trees

Predictive tools to distinguish between DF or DHF and chikungunya at presentation were constructed using classification and regression trees [27]. Classification and regression tree models are machine learning non-parametric techniques to classify categorical or continuous dependent variables as a function of multiple explanatory variables. The classification trees were constructed using a binary recursive partitioning algorithm for the elicitation of the rules using the tree package in R [28]. The trees were fitted to resourced-limited (clinical data only) and well-resourced settings (clinical and laboratory data) and then pruned using a cost-complexity measure to obtain the lowest cross-validated error. Leave-one-out cross-validation was employed to validate the decision trees and to estimate their predictive power [28]. The correct classification percentages for chikungunya and DF/DHF were then computed based on the classification as represented in the decision trees over the actual number of cases.

### Time course analysis

To quantify mean changes in temperature, serum hematocrit, platelet and leukocyte counts, while accounting for between patient variability, we used hierarchical modelling within the Bayesian framework. We used a Markov model for the daily means with homoskedastic stochastic innovations, homoskedastic errors, with random effects assumed to act multiplicatively on the grand mean. The data model is 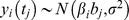, where 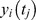 is the measurement of quantity *y* taken of individual *i* at time 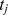 (if measured). The parameter model is 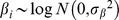, 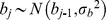 for *j*>1, 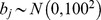 for *j* = 1 and 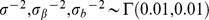. The model was fitted in openBUGS [29] using 100 000 iterations following 1000 iterations discarded as burn-in and with every tenth draw exported for subsequent analysis. Parameter estimates were then transformed to yield estimates and 95% credible intervals of the dynamic average values of the four covariates for both infections.

All statistical analyses were performed in the R statistical environment [29] or using openBUGS [30].

### Ethics approval

This study was approved by Domain Specific Review Board, National Healthcare Group, Singapore (DSRB-B/05/115 and DSRB-E/08/567).

## Results

### Differences in clinical expression between DF, DHF and chikungunya

**Figure 1:**
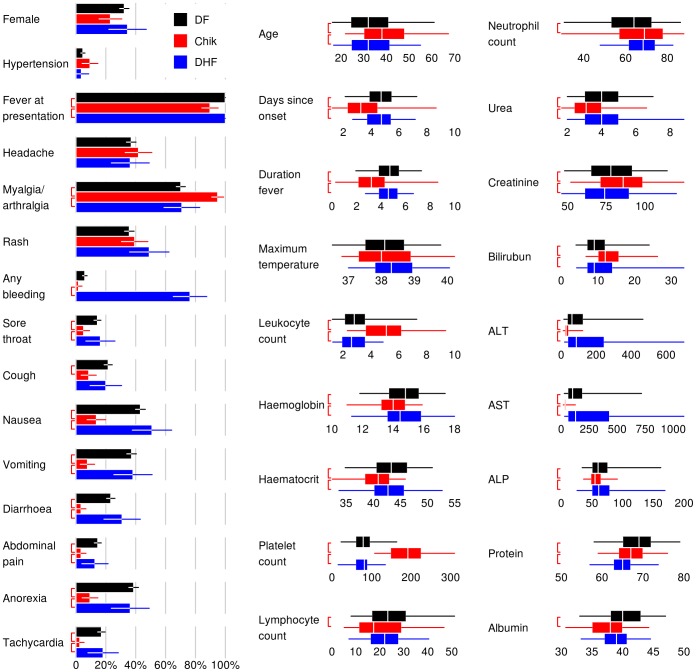
Univariate anaylsis of variables at first presentation to hospital. The analysis compared between Dengue fever (DF), dengue hemorraghic fever (DHF), and chikungunya (Chik). For binomial variables (first column on the left), bars denote mean percentage with whiskers denoting 95% confidence intervals. For continuous variables (right 2 columns), the box shows the median values (in white) with the interquartile ranges, while the whiskers denote the central 95^th^ percentiles. The red brackets to the left of the bars denote statistically significant comparisons between DF and chikungunya (upper brackers), and DHF and chikungunya (lower brackets). Uniformly distributed jitter of up to ±12 h has been added to the days since onset and duration of fever for graphical purposes. Five DF patients with no temperature measurement are excluded from the maximum temperature panel.

Differences between DF, DHF and chikungunya at presentation are shown in figure 1 (results over the course of hospitalization are similar and not presented). Not all chikungunya patients were febrile, although all dengue cases were. Statistically significantly more chikungunya patients had myalgia or arthralgia, and fewer had a sore throat, cough (for DF), nausea, vomiting, diarrhea, abdominal pain, anorexia or tachycardia than patients with DF or DHF. Notably, chikungunya patients had significantly higher leukocyte counts than either DF or DHF patients, with 76% of chikungunya cases having a leukocyte count of 3.6×10^9^/L or more, and 76–78% of DF and DHF patients having a leukocyte count of 3.6×10^9^/L or less. Stronger still was the difference between platelet counts with 92% of DHF and 77% of DF having a platelet count at presentation of <100×10^9^/L versus only 2% of chikungunya patients with similar degree of thrombocytopenia. However, there was substantial overlap in most signs, symptoms and laboratory measurements.

### Predicting chikungunya versus DF or DHF

The multivariate logistic regression analysis comparing DF versus chikungunya and DHF versus chikungunya at first presentation to hospital are shown in Tables 1a and 1b respectively. Tachycardia, cough, fever at presentation and duration, anorexia, and higher ALT, hematocrit, urea, and albumin levels were indicative of DF, while higher platelet count, hemoglobin, lymphocyte proportion, creatinine, bilirubin, and more myalgia/arthralgia were predictive of chikungunya. Bleeding, presence of fever and longer duration of illness at presentation were indicative of DHF, while a longer duration of fever and a higher platelet count increased the odds that the patient had chikungunya.

**Table 1:** Multivariate logistic regression of dengue fever versus chikungunya infection (Table 1a); and dengue hemorrhagic fever versus chikungunya (Table 1b) at presentation among in-patients Tan Tock Seng Hospital, Singapore.

Table 1a				
Variable	aOR	95% CI		p
Tachycardia (pulse>100/minute)	0.0029	0.00016	0.036	<0.0001
Cough	0.067	0.0052	0.59	0.012
Fever at presentation	0.042	0.0003	0.5	0.01
Serum alanine aminotransferase (per 100 units)	0.11	0.014	0.41	0.0001
Anorexia	0.14	0.023	0.67	0.011
Serum hematocrit	0.34	0.18	0.63	0.0003
Serum urea	0.35	0.2	0.57	<0.0001
Duration of fever (days)	0.6	0.39	0.89	0.0098
Serum albumin	0.71	0.6	0.83	<0.0001
*Leukocyte count*	1.53	1.06	2.22	0.022
*Platelet count (per 10 units)*	1.61	1.41	1.89	<0.0001
*Lymphocyte proportion (per 10 units)*	2.1	1.2	3.9	0.0063
*Serum creatinine (per 10 units)*	2.2	1.5	3.5	<0.0001
*Serum bilirubin (per 10 units)*	3.7	1.8	8.1	0.0011
*Myalgia/arthralgia*	13	3	73	0.0003
*Hemoglobin*	13	2.7	73	0.0013

Table 1b				
Variable	aOR	95%CI		p
Any bleeding	0.0001	0.0000	0.047	<0.0001
Fever at presentation	0.0002	0.0000	0.074	0.0005
Duration of illness (d)	0.086	0.0034	0.72	0.017
*Duration of fever (d)*	8.2	1.5	77	0.012
*Platelet count (per 10 units)*	9.1	2.1	5×10^8^	<0.0001

Estimates are derived using Firth's modified score procedure, and confidence intervals using profile penalised likelihoods, as described in the text. Adjusted odds ratios (aOR) are in favour of chikungunya infection: variables associated with chikungunya are indicated in italic type.

Over their entire hospital stay (Table 2a), DF patients were more likely to have tachycardia, fever, and higher ALT, urea and albumin levels, while chikungunya patients were more likely to have higher maximum creatinine, minimum neutrophil proportion, minimum platelet count, maximum temperature, and maximum bilirubin levels. DHF cases were more likely to have bleeding, fever, and tachycardia, while chikungunya cases were more likely to have higher minimum serum protein (Table 2b).

**Table 2:** Multivariate logistic regression of dengue fever versus chikungunya infection (Table 2a); and dengue hemorrhagic fever versus chikungunya (Table 2b) during entire hospital stay among inpatients at Tan Tock Seng Hospital, Singapore, 2006–8.

Table 2a				
Variable	aOR	95% CI		p
Tachycardia (pulse>100/minute)	0.00073	0.0000	0.027	<0.0001
Fever ever	0.0009	0.0000	0.048	0.0017
Maximum serum alanine aminotransferase (per 100 units)	0.4	0.018	0.66	0.0003
Maximum serum urea	0.51	0.29	0.8	0.002
Minimum serum albumin	0.65	0.5	0.79	<0.0001
*Maximum serum creatinine (per 10 units)*	1.78	1.27	2.69	0.0013
*Minimum neutrophil proportion (per 10 units)*	2.3	1.1	5.3	0.02
*Minimum platelet count (per 10 units)*	2.3	1.9	3.3	<0.0001
*Maximum temperature (°C)*	3.9	1.3	18	0.0094
*Maximum serum bilirubin (per 10 units)*	6.1	2	19	0.0022

Table 2b				
Variable	aOR	95% CI		p
Any bleeding ever	0.000053	0.0000	0.0017	<0.0001
Fever ever	0.009	0.000034	0.34	0.014
Tachycardia (pulse>100/minute) ever	0.029	0.0009	0.46	0.011
*Minimum serum protein (per 10 units)*	43	4.7	2000	0.0003

Estimates are derived using Firth's modified score procedure, and confidence intervals using profile penalised likelihoods, as described in the text. Adjusted odds ratios (aOR) are in favour of chikungunya infection: variables associated with chikungunya are indicated in italic type.

**Figure 2:**
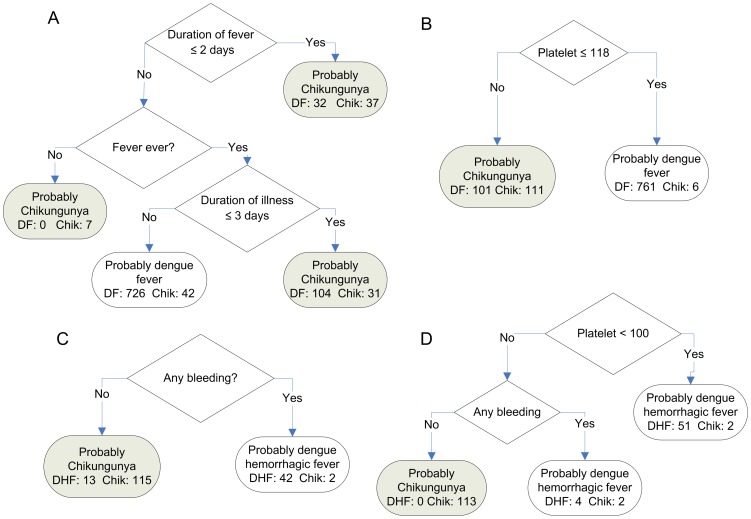
Decision tree models for discrimination. Models discriminate between dengue fever (DF) or dengue hemorrhagic fever (DHF) and chikungunya (Chik) for well-resourced (laboratory data included) and resource-limited (laboratory data excluded) settings. A and B discriminate between chikungunya and DF in a resource-limited and well-resourced setting respectively. C and D discriminate between chikungunya and DHF in a resource-limited and well-resourced setting respectively. Final classifications as chikungunya are shaded in grey, while classifications for DF/DHF are unshaded.

The decision trees for determining DF versus chikungunya, and DHF versus chikungunya at first presentation to hospital are shown in Figure 2. The tree designed for resource-limited settings without laboratory testing comparing DF and chikungunya (Figure 2A) presented sensitivity of 95% and specificity of 36% in the prediction of dengue fever (Table 3) but was a relatively poor predictor of chikungunya (64% positive predictive value, Table 3). If laboratory variables are incorporated, the sensitivity increases by 4% and the specificity by 16% and 32% for the prediction of DF and chikungunya respectively (Table 3). The tree comparing DHF and chikungunya in a limited-resource setting (Figure 2C), has high sensitivities and specificities but a positive predictive value of only 76% (Table 3). The tree for DHF versus chikungunya using laboratory variables (Figure 2D) could identify all DHF patients correctly (positive predictive value of 100%) and almost all the chikungunya patients (positive predictive value of 97%, Table 3). The trees could discriminate very well between chikungunya and dengue with a single laboratory variable: the platelet count. Without laboratory variables it is still possible to discriminate reasonably well for DHF (using the rule, bleeding implies DHF). However, the use of this tree as an admission protocol in resource-limited settings might be questionable since 24% of DHF patients would not be hospitalized and this may have severe clinical implications (positive predictive value of 76%, Table 3). The tree we identified as best for distinguishing DF and chikungunya relied on the duration of illness and fever which was not very discriminating, indicating the difficulty in characterizing these two illnesses solely with signs or symptoms (Figure 2A).

**Table 3:** Sensitivity (sens.), specificity (spec.), positive predictive value (PPV) and area under the receiver operating curve (AUC) for decision tree models to discriminate between dengue fever (DF) or dengue hemorrhagic fever (DHF) versus chikungunya, using data at presentation.

Model	Suitable for less resourced settings?	Sens. detect DF/DHF	Spec. detect DF/DHF	PPV detect DF/DHF	Sens. detect chikungunya	Spec. detect chikungunya	PPV detect chikungunya	AUC
chikungunya vs DF	Yes	0.95	0.36	0.84	0.36	0.95	0.64	0.59
chikungunya vs DF	No	0.99	0.52	0.88	0.52	0.99	0.95	0.91
chikungunya vs DHF	Yes	0.95	0.90	0.76	0.90	0.95	0.98	0.91
chikungunya vs DHF	No	0.93	1.00	1.00	1.00	0.93	0.97	0.99

### Temporal trend of DF, DHF and chikungunya

**Figure 3:**
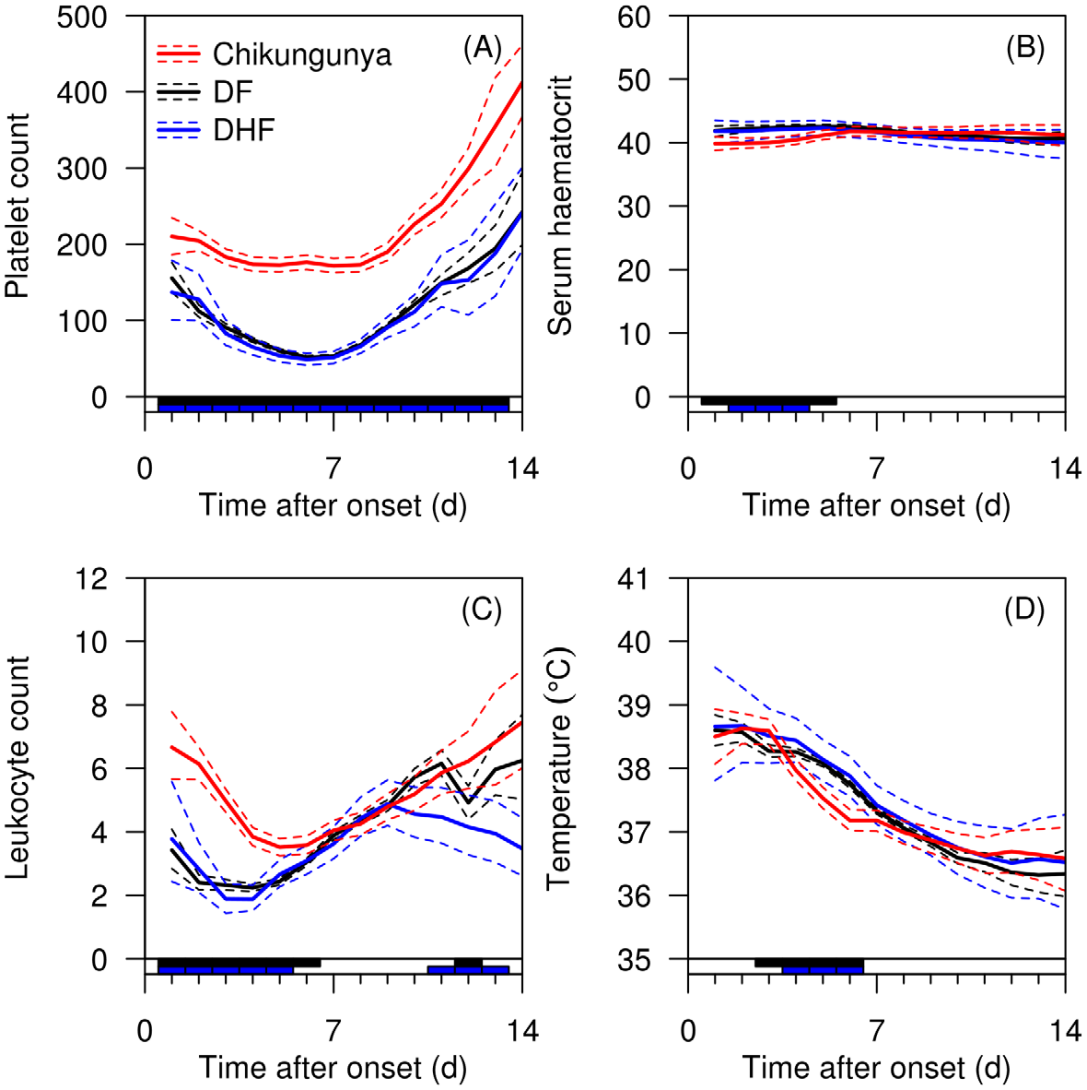
Time course analysis of selected variables. Analysis shows platelet counts (A), serum hematocrit (B), leukocyte (C), and temperature (D) for dengue fever (DF), dengue hemorrhagic fever (DHF) and chikungunya. Individual data are indicated in semi-transparent red (chikungunya), black (DF), and blue(DHF) lines. Overall means are indicated as solid lines, with 95% credible intervals as dashed lines. The bar on X-axis indicates in black days with a ‘significant’ difference (defined as 95% credible interval for the difference between the two disease means not crossing zero) between chikungunya and DF, and the blue bar between chikungunya and DHF.

The time course analysis presented in Figure 3 supports platelet count as the key distinguishing variable for chikungunya and dengue infections, with the average platelet count scarcely dropping below 200×10^9^/L in patients with chikungunya, but dropping below 100×10^9^/L in dengue infections. Chikungunya caused a slower drop in leukocyte count than dengue infections. Smaller differences were present for hematocrit and temperature.

## Discussion

Chikungunya and dengue share overlapping geographic range and competent vectors [3], [31], with chikungunya occurring in epidemics in Africa, India, Sri Lanka and Southeast Asia [3], and dengue in most tropical and subtropical regions of the world [32]. Both have caused autochthonous outbreaks in non-endemic areas, namely dengue in Hawaii and Texas-Mexico border [33] and metropolitan France [34], and chikungunya in Italy [35] and metropolitan France [34]. Notably, serological surveys in Southeast Asia documented the presence of both dengue and chikungunya [36]; and similar findings were present among German aid workers returning from Benin, Burkina Faso, Cameroon and Thailand [37]. Concurrent dengue and chikungunya may also occur, as proven by PCR in 10 of 38 patients in Madagascar [38] and in a traveller to Singapore [39].

While chikungunya has occurred in localized epidemics in in Africa, South and Southeast Asia since the 1950's, a molecular mutation A226V resulting in more efficient viral replication and transmission in *Aedes albopictus* has enabled rapid expansion of its geographical range in the Indian Ocean since 2006 [40]–[42]. The overlapping geographic range and clinical manifestations of chikungunya and dengue, often in resource-limited countries, has made a diagnostic tool utilizing simple clinical criteria relevant and potentially useful.

In our study, there was substantial overlap in the symptoms and signs for dengue and chikungunya infections although key significant differences existed. Many of these differences have substantial overlap and there is substantial variability between individuals with the same illness, rendering their utility in diagnostic differentiation limited (Figure 1). Similar to previous studies [11]–[17], the differences that are most apparent at presentation are leukocytosis and myalgia/arthralgia which were more likely to be present in chikungunya cases, while thrombocytopenia were more likely to be present in dengue cases. In addition, during the entire course of illness, thrombocytopenia and neutropenia were more likely to be present in dengue cases. To assist doctors in differentiating between these two infections for appropriate triaging for site of care and clinical management, diagnostic and prognostic algorithms that are highly sensitive with high negative predictive values are desirable. Specifically, there is a need to identify patients with DHF as they require meticulous follow up and clinical management in hospital, while uncomplicated dengue and chikungunya can be managed on an outpatient basis.

The decision trees shown in Figure 2 will provide doctors with the necessary tools to identify DF versus chikungunya and more importantly DHF versus chikungunya. These simple tools can also supplement other laboratory tools such as the rapid immunochromatographic NS-1 tests for dengue which are now available and can provide additional differentiation between these two diseases [43]. Using only clinical variables of fever, duration of fever and illness (Figure 2A, likely to be only parameters available in resource-limited settings) is not as discriminating as the single laboratory variable of platelet count (Figure 2B) for DF versus chikungunya (Table 3). Similarly, the decision trees comparing DHF versus chikungunya in resource-limited settings (using bleeding only, Figure 2C) would incorrectly classify 24% of DHF cases which may lead to inappropriate case management. In well-resourced (using platelet count and bleeding, Figure 2D) settings, discrimination for DHF is 100%. For both DF and DHF versus chikungunya, decision trees using clinical variables only performed less well compared with clinical and laboratory variables (area under the receiver operating curve [AUC] for DF versus chikungunya, 0.59 versus 0.91, and for DHF versus chikungunya, 0.91 versus 0.99).

While it is useful to examine these variables at a static time point especially at hospital presentation, it may be helpful to understand the development of key clinical variables across time as the time of presentation to healthcare settings may vary in different settings. From our time course analysis, it is evident that DF and DHF had significantly lower platelet count across the entire hospitalization while the mean platelet count in chikungunya was within the normal range. Notably, DF and DHF had significantly higher hematocrit and temperature in the first week of illness, as rising hematocrit represents plasma leakage, a hallmark of DHF [5], [6]. Interestingly DF and DHF had significantly lower leukocyte count in the first week of illness, noted in similar work in Vietnam and Singapore [44]; this recovered during the start of the second week.

There are some limitations to our study. The dengue virus predominant in 2004 was serotype 1, and our cohorts comprised adult patients. There is a need to validate our findings in different settings with different dengue serotypes and children for better generalization. In addition, our dengue and chikungunya cohorts were from different time periods. Although the management protocol for dengue did not change substantially from 2004 to 2008, our chikungunya cases occurred during the first ever large-scale outbreak in Singapore with heightened national alert for clinical case detection. This would affect time from illness onset to presentation (days since onset, Figure 1). The clinical and laboratory data for chikungunya were collected prospectively during the outbreak, while the data for dengue were retrospectively collected. However, all our dengue cases were managed by doctors experienced in dengue treatment in the Department of Infectious Diseases with a standardized care path, which mitigated somewhat potential data inaccuracy in a retrospective study. The number of chikungunya and DHF cases in this study was relatively small, and although key variables could still be estimated accurately, future studies should be performed with larger datasets in different settings to validate these findings. In addition, while the 1997 WHO criteria was used to classify dengue cases, future studies should also consider the 2009 WHO criteria for severe dengue classification for comparison.

Dengue and chikungunya infections continue to co-exist in many tropical countries. Our study has shown that there is indeed substantial overlap in clinical presentation between these infections. At the same time, we have also shown that it is possible for clinicians to use simple clinical and laboratory variables to predict these infections for appropriate management.
